# The design and use of ACASI to elicit sexual behaviour in the PHASE survey – the Gambian experience

**DOI:** 10.1186/s12978-026-02339-4

**Published:** 2026-05-05

**Authors:** Adedapo O. Bashorun, Larry Kotei, Ousubie Jawla, Solinda Gomez, Sheikh-Omar Jallow, Nfamara Camara, Mehrab Karim, Mamadou SK. Jallow, Abdoulie F. Jallow, Emmanuel U. Richard-Ugwuadu, Bai Lamin Dondeh, Ed Clarke

**Affiliations:** Vaccines and Immunity Theme, Medical Research Council Unit at London, School of Hygiene and Tropical Medicine, Fajara, The Gambia

**Keywords:** Sexual behaviour, Human papillomavirus(HPV), Audio-Computer-Assisted Self-Interviewing (ACASI), Community advisory board (CAB), Cultural adaptation, Sub-Saharan Africa, Sexually-transmitted infections (STIs), And low-literacy settings

## Abstract

Collecting reliable sexual behaviour data remains a major challenge in conservative, low-literacy contexts such as The Gambia, where stigma, privacy concerns, and social desirability bias influence responses. The Prevalence of Human Papillomavirus, Antimicrobial resistance, and Sexually transmitted infection Estimation (PHASE) survey sought to address this challenge by introducing Audio Computer-Assisted Self-Interviewing (ACASI) for the first time in The Gambia. This paper documents the design, development, and piloting of the ACASI tool, highlighting key lessons for similar settings in sub-Saharan Africa.

A Community Advisory Board (CAB) was central to the tool’s development, ensuring cultural sensitivity, acceptability, and linguistic accuracy. CAB members guided the drafting and adaptation of sexual behaviour questions, advised on culturally relevant response categories, and reviewed translations into four local languages (Mandinka, Serahule, Fula, Wolof). They also oversaw the design of non-threatening, familiar images to accompany response options and ensured accurate, context-appropriate audio recordings. Technical implementation was achieved using the SurveyCTO platform, allowing offline use, encryption, and integration of multilingual audio and images. Privacy safeguards were embedded, including screen codes replacing text to prevent disclosure in shared settings and restricted staff access to individual responses.

Pilot testing revealed critical operational considerations. Many women lacked prior experience with digital tools and required extra support during the introductory phase. Adjustments included re-recording unclear questions, reordering items to reduce priming effects, and randomising response lists to enhance validity. Additional measures, such as repeating select questions at different points, improved internal consistency checks. Despite these challenges, participants demonstrated increasing confidence with the tool, and CAB feedback confirmed that ACASI was more acceptable for sensitive questions than face-to-face interviewing.

Our experience demonstrates that ACASI is a feasible and culturally appropriate method to collect sexual behaviour data in The Gambia, even within low-literacy, conservative populations. The use of familiar images, validated translations, and community engagement enhanced usability, while technical refinements addressed privacy and confidentiality concerns. Key challenges related to computer literacy and dialect variation were addressed through training, piloting, and iterative refinement. This process illustrates that by prioritizing community involvement, technical adaptation, and cultural sensitivity, ACASI offers a promising framework to enhance the acceptability and potentially the accuracy of sexual behaviour reporting in large-scale epidemiological surveys. These lessons can be applied to similar sub-Saharan African contexts seeking to generate high-quality data to inform HPV (human papillomavirus), STI (sexually transmitted infection), and sexual health research.

## Background

Eliciting reliable data on sexual behaviour is challenging because the factors influencing these behaviours are complex, difficult to measure objectively, and often private, making direct observation unethical or impractical [1, 2]. It is well documented that risky sexual behaviour poses a significant global public health challenge because it contributes to serious health outcomes, including cervical cancer, stillbirths, HIV/AIDS, and infertility. In many regions, discussions about sexual behaviour are regarded as taboo or private, which, in research, often leads to underreporting [3] or social desirability bias [4] —where participants provide socially acceptable responses instead of truthful ones. In The Gambia, conversations around sexual behaviour are infrequent, as they are typically associated with promiscuity. Women, in particular, who engage in such discussions are frequently subjected to stigma [5]. Asking people face-to-face about their sexual behaviour – particularly outside a medical context—is widely regarded as culturally inappropriate and disrespectful.

Differences in literacy and understanding of sexual behaviour terms also complicate data collection, particularly in low- and middle-income countries. Also, religious and cultural beliefs, and the legal system in these countries, determine how questions regarding sexual behaviours are framed and interpreted. Privacy concerns, stigma, and fear of judgment may hinder accurate reporting, especially in conservative societies like The Gambia, where non-traditional behaviours are condemned. Lastly, lack of standardized methodologies, inconsistent definitions and technological barriers in survey administration add to the difficulty of measuring reliable data on sexual behaviour across diverse global contexts. In recent years, the use of the Audio Computer-Assisted Self-interviewing (ACASI) has been employed to address some of these challenges. ACASI is a method of data collection used in research, surveys, and assessments that combines computer technology with audio recordings to facilitate self-administered interviews [2, 6–11]. In ACASI, the questions are pre-recorded on a computer or tablet, and the participant listens to the questions through headphones and selects their answers via a touchscreen or keyboard. This can involve pressing buttons on the screen, using a mouse, or typing [9]. ACASI has been employed mostly in HIV behavioural research and studies examining the intersection of sexual violence, refugee status, and criminal behaviours, in which the study participants will be uncomfortable giving accurate answers to the interviewers when sensitive questions are asked [8]. Although ACASI was initially designed and widely used in high-income countries, its adoption in low- and middle-income countries (LMICs) has remained limited [1, 6, 7, 12, 13]. The few studies done in LMICs show that ACASI improves the accuracy of sexual behaviour reporting, increases participants’ privacy and reduces social desirability bias by eliminating the potential influence an interviewer may have on the respondent’s answers [2, 12, 14]. They also suggest that ACASI can be suitable in low-literacy settings like ours. In a close-knit society like The Gambia, where even in clinical settings, it can be difficult for patients to disclose risky sexual behaviour to their caregivers due to fear of being exposed or judged or stigmatised, ACASI can be a valuable tool to use in surveys.

The PHASE (Prevalence of Human Papillomavirus (HPV), Antimicrobial resistance (AMR), and Sexually Transmitted Infections (STI) Estimation Survey) survey aims to quantify the effects of sexual behaviours on HPV prevalence [15]. A key element of this includes understanding how sexual mixing patterns impact these prevalence estimates. This will require the survey team to ask participants sensitive questions regarding sexual behaviours, including outside marriage, which may be perceived as socially undesirable in The Gambia. Therefore, an ACASI tool was designed to assess the sexual behaviour of the participants in the PHASE survey instead of the traditional face-to-face interviewing method.

This approach is expected to be more culturally acceptable and likely to improve reporting accuracy, based on findings from similar studies [1, 2, 13]. To our knowledge, this will be the first time ACASI will be employed in The Gambia, and used in an STI prevalence survey of this magnitude in West Africa. This article is to document our experience from the design to the implementation stage of this tool in The Gambia.

## Summary of the PHASE survey

The PHASE survey is a cross-sectional, population-based, probability proportional to size, multistage cluster survey aimed at estimating the prevalence of HPV, AMR and STI in a minimum of 10,230 females aged 15 to 49 years in The Gambia [15]. The Gambia, located within West Africa, is one of the world’s poorest countries, largely dependent on agriculture, with more than 40% of the population living in poverty [16]. It has a population of about 2.4 million people [17], predominantly Muslims (96%) and only 47% of women aged 15 to 49 years are literate [16]. The PHASE survey is being conducted in the south bank of the upper river region of The Gambia using the Basse Health Demographic Surveillance System as the sampling frame [15]. The Basse LGA (local government area), or the upper river region, is the third largest local government area in the country, accounting for 10.8% of the country’s population [17]. The sampling strategy will account for clustering at district, village, compound and household levels. The south bank of the Basse LGA of The Gambia consists of four districts – Kantora, Tumana, Basse, and Jimara- and four languages are predominantly spoken in this region: Mandinka, Serahule, Fula, and Wolof. These languages are not widely written, and with an abysmally low English literacy rate of 19% [16] in the Basse LGA, making written questions on a computer or tablet inappropriate.

The main objective of the survey is to establish the prevalence of HPV infection through the measurement of urinary HPV-DNA and to quantify the effects of sexual behaviours, the presence of other STIs (HIV, Hepatitis B and C, Syphilis, Gonorrhoea, Chlamydia, Mycoplasma, Trichomonas), gynaecological history, cervical cancer screening and other sociodemographic characteristics on HPV prevalence. HIV, Hepatitis B and C, Syphilis were detected through venous blood sampling using WHO-prequalified point-of-care test kits, while Gonorrhoea, Chlamydia, Mycoplasma, and Trichomonas were detected using the urine via a real time PCR assay (The commercial Allplex™ STI Essential Assay). Full details are in the published PHASE study protocol [15].

We also aim to measure the sexual mixing patterns within our study population. This area has received little attention in STI transmission research, which has more often focused on the distribution of sexual behaviours within the population [18]. Gaining insights into sexual mixing patterns is critical in better understanding the dynamics of STIs, including their acquisition and spread [18]. In our context, with an estimated polygamy rate of 34%, [16] understanding these patterns becomes even more valuable.

## Development of the ACASI

A community advisory board (CAB) was established to guide the design and implementation of the entire survey, including the development of the ACASI tool. The involvement of the CAB was instrumental in ensuring that the tool reflected community perspectives and cultural appropriateness in its design and content.

### Formation of the community advisory board

We ensured that the CAB was truly representative of the community and population (female) in which we are going to conduct the survey through the deliberate design of the membership criteria for each of the two groups – female and male.

For the female group, members were selected by randomly choosing three villages per district, taking ethnicity into account. In each selected village, the head (Alkalo) and the women’s leader were approached to nominate two females after we explained the structure and purpose of the study, and one female was then randomly chosen by ballot. The eligible female had to be a resident in the village, have extensive knowledge of the village and its language, and be willing to attend meetings. It was essential (as per customs) that the husband or household head consented to the nominated female’s participation; otherwise, another female was selected from the same village. English literacy was not required, as we sought the perspective of laypersons, whose backgrounds reflected those of the study population. The female group consisted of 12 members.

For the male group, one representative was selected from each district following recommendations from community stakeholders who possessed substantial knowledge of local community dynamics. The male must be a resident of the village with experience or knowledge about research projects. The 4-member male group was intended to complement the perspectives of the female group.

### Drafting of the questions

A literature review was conducted to identify exposures and characteristics relevant to HPV prevalence, particularly in low- and middle-income countries. Searches were done in the following electronic databases: PubMed, Scopus and Google Scholar.

The search terms used included combinations of:

- “HPV prevalence” OR “human papillomavirus infection”
- AND “risk factors” OR “sexual behaviour” OR “reproductive health”
- AND “questionnaire” OR “survey tool” OR “data collection”
- AND “low- and middle-income countries” OR “sub-Saharan Africa”.

Boolean operators (AND, OR) were used to refine the search, with English-language materials prioritised.

In addition to published studies, our team consulted experienced researchers who had previously conducted sexual and reproductive health surveys in similar sociocultural settings to obtain copies of their survey tools. Findings from the published and grey literature, along with available example questionnaires, were compiled, leading to the development of the first draft of the questions to be included in the ACASI.

### Review of the draft questions

The draft questions underwent several rounds of review, with significant input from the CAB. The process began with the CAB, evaluating the first draft for appropriateness and relevance.

Following this, we explored additional sexual behavioural exposures and characteristics specific to the region. The CAB played a crucial role in this review, removing culturally inappropriate or hard-to-translate questions to respect the different cultures within the region. Some notable examples were:

- In the question on material support for sexual intercourse, the CAB recommended including mobile phones, clothes, jobs, or the opportunity to travel abroad in the list of potential answers, as these are common incentives men use in the community to lure women for sexual intercourse.
- We initially wanted to assess participants’ knowledge about the types of STIs, but the CAB advised removing this question. They explained it would be too difficult for our participants to answer, and also, the STI terms might be ambiguous when translated into local languages. Instead, they recommended asking about STI symptoms, which most women in the region will be familiar with.
  While we incorporated much of their advice, we chose not to include others. For example –
- The CAB highlighted a reported but poorly documented practice among women whose husbands are abroad, involving the insertion of unspecified local substances into the vagina to enhance sexual pleasure. They recommended including a question on this behaviour in the survey. However, because no member could provide details on the exact substances used, and their scientific relevance could not be assessed, this was not incorporated. We believe this should be further explored in another study specifically designed for that purpose. This decision was explained to the CAB, who accepted the rationale.

Subsequently, we reviewed the questions with other relevant stakeholders, experienced researchers, and field experts, followed by an internal review by our team. This process spanned several months and involved multiple meetings with the CAB. The final draft of the questions was reviewed and agreed upon with the CAB before submission for ethical approval.

### Image development

**Fig. 1:**
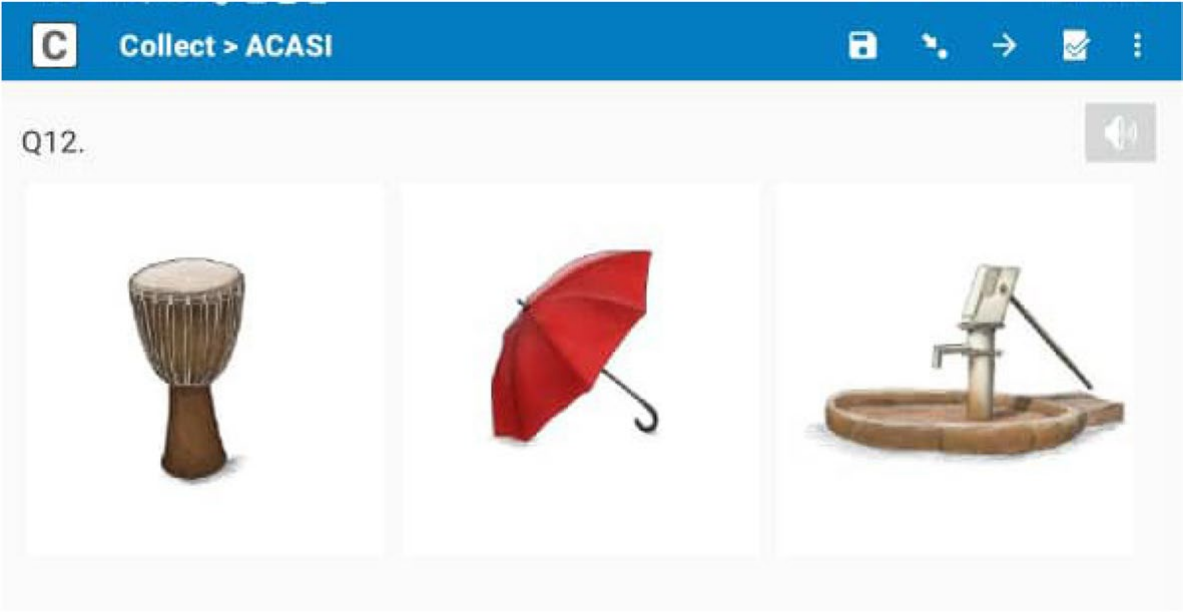
snapshot of question 12 on the mobile tablet

**Fig. 2:**
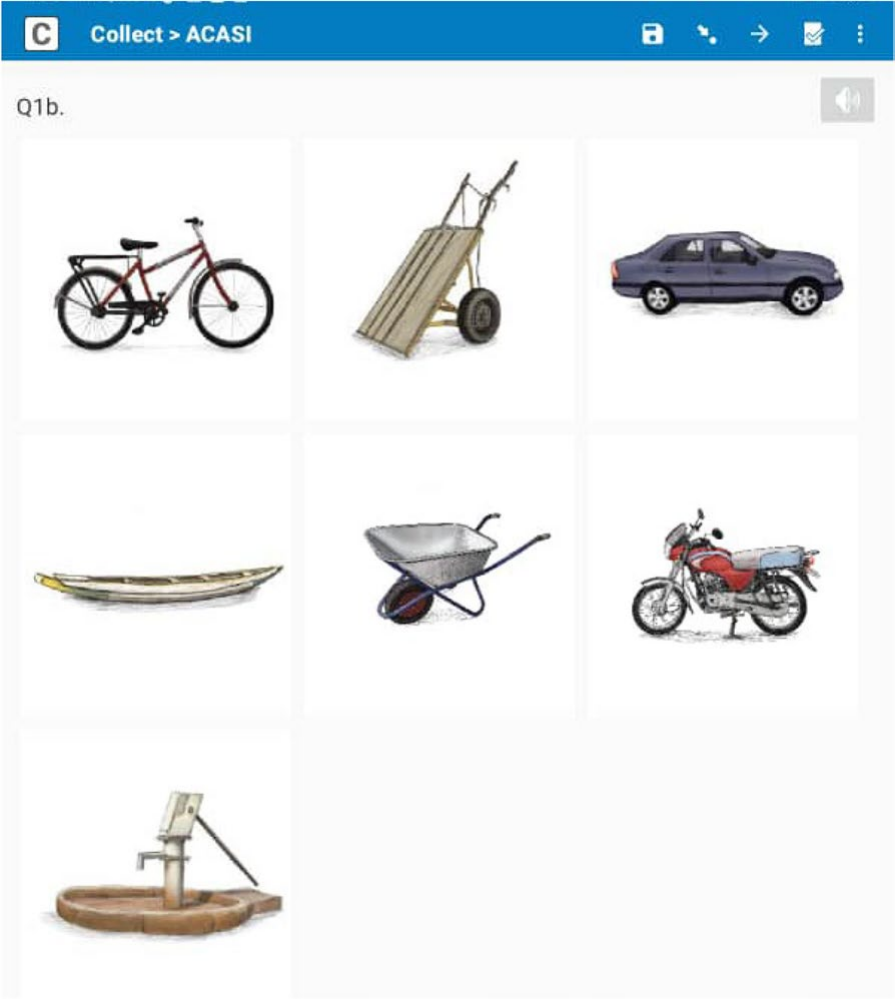
snapshot of question 1b on the mobile tablet

**Fig. 3:**
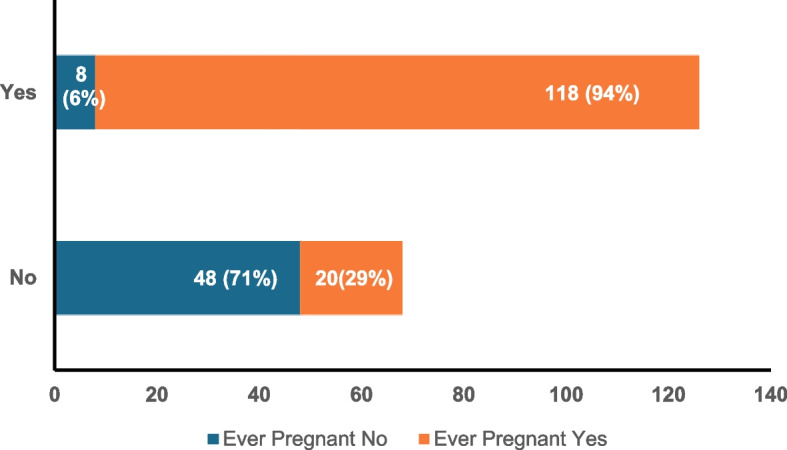
Ever had sexual intercourse history

All the images used were from a list of suggestions by the CAB members. These were images of common items familiar to people, especially females, in the study area and do not evoke fear or negative emotions. Examples are – ataya (popular tea in The Gambia) pot, motorcycle, local borehole hand pump, okra, goat, donkey cart, pepper, bicycle, wheelbarrow, etc., as shown in Figs. 1, 2 and 3.

These images did not portray any relationship to sexual behaviour or insinuate the answer to a particular question, which was critical in protecting confidentiality and improving the acceptability of the ACASI tool. The selected items were submitted to a graphic artist familiar with The Gambia and the Basse LGA who created drawings of each item.

The initial drafts of the drawings were reviewed by our field team, and any image that did not accurately represent the intended item was sent back for revision. Once our team was satisfied with the accuracy of the drawings, the subsequent drafts were reviewed by the CAB to ensure that each image was easily recognizable. Any potentially conflicting images were either replaced or returned to the graphic artist for further adjustments.

All images featured a plain white background, with each image painted in its respective colours (e.g., a pepper was painted red). Extreme care was taken to avoid using colours that might be naturally associated with positive or negative emotions or explicitly represent the colour of any political party.

### Language selection and validation

The language selection process for the ACASI was guided by the ethnic composition of the tribes in the south bank of the Upper River Region (URR) of The Gambia. The primary ethnic groups in this area are Serahule, Fula, and Mandinka; with Wolof also included due to the presence of migrants in Basse from other parts of The Gambia and Senegal.

The selection was based on the understanding that participants would likely speak and understand at least one of these four languages or English, the official language of The Gambia.

Major considerations were made regarding dialect variations during language selection. For example, there are about four major Fula dialects spoken within the study area, and although every Fula person has a general understanding of each dialect, there are subtle differences in terminologies regarding sexual behaviour. This was particularly evident when Fula women in the CAB discussed the choice of terminologies for the questionnaire. We observed misunderstanding of some of the sexual behavioural terms across the different Fula dialects. Thus, the CAB advised us to select the dialect understood by most Fula’s within the region. At some points during translation, certain words and phrases from other Fula dialects were imported into our translations because they were more widely understood and conveyed exactly what the survey team were asking. This blend was necessary for increasing the understanding amongst the four Fula dialects.

The CAB played a crucial role, offering guidance on appropriate dialects and helping ensure translations were culturally and religiously sensitive. The understanding behind each question was discussed in focus groups with the CAB, ensuring clarity and cultural appropriateness.

Translations from English into the local languages involved numerous meetings to ensure questions retained their original meaning, were culturally sensitive, and easily understandable. Survey team members who spoke the local languages facilitated discussions to clarify the questionnaire's intent. CAB members fluent in each language unanimously agreed upon translations. To maximise consistency of understanding, the final agreed translations with the CAB were audio-recorded for referencing while making the audio recordings.

### Audio recordings

The recording process required extensive preparation and close collaboration with the CAB. To ensure linguistic accuracy, sub-groups within the CAB were organised by native language to oversee the process. Five women with clear and articulate voices were selected for recording, following preparatory meetings and rehearsals, with the final sessions conducted in a studio. The CAB provided real-time feedback during recordings to guarantee both accuracy and cultural appropriateness. Considerable discussion was needed to identify suitable local-language equivalents for English words and phrases, and to capture cultural nuances in speech that could otherwise lead to misinterpretation. Particular attention was given to intonation, emphasis, and pauses between words to maximise comprehension. For example, CAB members rejected the first woman chosen to record the Fula version, as they judged her intonation and emphasis inadequate, despite her fluency in the preferred dialect (Fula Kunda).

### Confirming the accuracy of audio recordings

Before proceeding with the technical design, the audio recordings in local languages were carefully reviewed to ensure they accurately translated the questions from the English version.

This involved testing the ACASI with CAB members from various linguistic backgrounds to gather feedback. Our team as well as two independent translating agencies verified the audio recordings through back-translation into English. As such, two back-translations each were provided by independent translators per language. Only slight adjustments were made after this process, with most of the questions being accurately back-translated into English.

## Technical design

Our ACASI tool was developed using the SurveyCTO (Survey Collect, Transform, and Optimize) platform, a mobile data collection system designed specifically for field settings, particularly in locations (like ours) where devices may not always have internet access [19]. The SurveyCTO platform has been successfully used to design the ACASI tool in similar settings, especially in modern slavery studies [19]. The ACASI tool is a significant advancement in survey methodology, empowering respondents to interact with survey questions through a digital interface, using audio to guide them throughout the process. This section explores the key features and considerations in developing the ACASI tool for this survey.

### Developing a demo version

Our initial task was to create a demo version using random images, questions, and responses. A team member recorded a few questions and responses in English using a mobile phone. This enabled evaluation of the tool’s functionality and compatibility with the Medical Research Council Unit The Gambia at London School of Hygiene and Tropical Medicine server. Having built a successful concept, we then embarked on full ACASI tool design for the survey’s specific needs.

### Building the ACASI form

After confirmation of the final approved questionnaire, SurveyCTO’s Form builder was used by our data team to create the draft survey questions. At this point, we were able to customize the form by adding relevant constraints and creating lists of choices. Thanks to SurveyCTO’s multi-language capacity, we were able to offer the survey in English language and 4 local dialects—Mandinka, Serahule, Fula and Wolof—so respondents could choose the language they preferred at the beginning of the survey. Transitioning to the next question automatically after a response was a major user requirement from the survey team. Since this feature is not supported by the default settings of SurveyCTO, we built a custom plug-in to enable this functionality.

### Uploading the images and audio recordings

All images and audio recordings were tagged appropriately for easy identification. The images, in JPEG format, and the audio recordings, in MP3 format, were uploaded to the media library of the SurveyCTO database. Each image was embedded into the corresponding choice lists, and the audio recordings were integrated into each question for the five different languages. The audio files were configured to autoplay when the field is first accessed. Once the image and audio file integration was complete, both the data and survey teams tested them to ensure correct integration and functionality in both the online and offline versions.

### Data privacy and security

A fundamental design component of the ACASI tool was keeping data private and secure. The following steps were therefore taken to maintain data confidentiality:

- Data encryption: The tool is designed with end-to-end encryption, ensuring data is always protected from unauthorised access, be it at rest or in transit.
- Anonymization techniques: Each participant was identified with a unique survey number, and no personal identifiers were included in the ACASI.
- Access control: Access to the dataset was restricted only to authorised personnel, who were mostly the clinical staff of the survey team.

## Final validation and confidentiality measures of the ACASI tool

Following the development of the ACASI database, the survey team conducted a series of test runs—we term them “dry runs”—with members of the CAB. These sessions evaluated the tool’s user-friendliness, clarity, and ability to uphold participant confidentiality.

Based on feedback from these assessments, several revisions were implemented to strengthen both usability and privacy:

1. Strengthened Confidentiality Messaging: The CAB strongly recommended reinforcing the confidentiality of participants’ responses within the ACASI introduction. Although this assurance would have already been communicated verbally by survey staff, embedding it directly in the ACASI script served to further reassure participants.
2. Screen Privacy safeguards: During testing, it became apparent that visible question text and response options could potentially compromise privacy in less controlled settings. To address this, all questions and responses were replaced with alphanumeric codes as shown in Figs. 1 and 2. This measure ensures that even if someone—such as a parent or passerby—glances at the screen, they cannot infer the content, thereby protecting participant confidentiality.
3. Restricted access for Field staff: We ensured that at the end of the ACASI administration, field staff do not have access to the responses except for a notification that indicates that the respondent had completed the ACASI, further safeguarding participant privacy.

## Sections of the ACASI

### Introduction

This section introduced participants to the ACASI system using non-sensitive questions, such as marital status. These questions served a dual purpose: first, to familiarise participants with the tool and ensure they could navigate it confidently; and second, to cross-validate responses previously collected during the face-to-face interview. Cross-validation was important for identifying inconsistencies, clarifying potential misunderstandings, and strengthening the reliability of the data. A field staff member guided each participant through this section, confirming comprehension before moving forward. Headphones were not used at this stage.

### The questionnaire

This section consists of the sexual behavioural questions. A headphone is provided, and the participants complete this section independently in a private area.

### Closing

This final section informs the participant that the questionnaire is complete, expresses appreciation for her participation, and provides instructions to return the tablet to the designated field staff member.

## Piloting

The pilot, including all survey procedures along with the ACASI tool, was conducted in a small village that was not among those randomly selected for the main survey. The primary objective was to further assess participants’ ability to use the ACASI tool on tablet computers and to ensure the questions were consistent and clear.

### Pilot results

A total of 15 women were surveyed. Most (80%; 12/15) experienced difficulty using the tablet device, and one response was missing due to technical issues. The majority were married (93%), had no formal education (53%), and were of Fula tribe (79%). Other sociodemographic characteristics are shown in Table 1.

**Table 1:** Sociodemographic characteristics

Variable	Grouping	Frequency (*N* = 15)	Percentage (%)
Residency duration	1–5 years	6	42.86%
	> 5 years	8	57.14%
Age Group	15-29yrs	7	50.00%
	30-49yrs	7	50.00%
Marital Status	Married	13	92.86%
	Widowed	1	7.14%
Education	No formal	8	53.33%
	Primary	3	20.00%
	Secondary	3	20.00%
	Post Secondary	0	0.00%
Employment	Unemployed	14	100.00%
Tribe	Fula	11	78.57%
	Mandinka	2	14.29%
	Serahule	1	7.14%

A majority (79%) reported being sexually active, with 91% of these individuals initiating sex between the ages of 18 and 20 years. Sexual history (shown in Table 2) was characterised by high stability, as 91% reported a spousal first partner and 100% identified their husband as their only lifetime sexual partner. Regarding sexual practices, oral sex was reported by 21% of respondents, while the vast majority (91%) reported never having engaged in anal sex. Notably, the assessment of sensitive behaviours showed high engagement with the tool, although 18% of participants chose not to disclose their history regarding forced sex.

**Table 2:** Some Sexual behavioural characteristics of the pilot participants

Variable	Grouping	Frequency (n)	Percentage (%)
Ever had Sex	No	3	21.43%
	Yes	11	78.57%
	Total	14	100.00%
Age at First Sex	15 to 17 years	1	9.09%
	18 to 20 years	10	90.91%
	Total	11	100.00%
First Sexual Partner	Non-spousal	1	9.09%
	Spousal	10	90.91%
	Total	11	100.00%
Number of men ever slept with	1	10	90.91%
	2	1	9.09%
	Total	11	100.00%
Age category of men ever slept with	Always older	8	72.73%
	Mostly older	2	18.18%
	Always younger	1	9.09%
	Total	11	100.00%
Lifetime Sexual Partners	Husband	11	100.00%
Oral Sex History	Never	11	78.57%
	A few times	3	21.43%
	Many times	0	0.00%
	Prefer not to answer/cannot remember	0	0.00%
	Total	14	100.00%
Anal Sex History	Never	10	90.91%
	A few times	0	0.00%
	Many times	0	0.00%
	Prefer not to answer/cannot remember	1	9.09%
	Total	11	100.00%
History of Forced Sex	Never	9	81.82%
	A few times	0	0.00%
	Many times	0	0.00%
	Prefer not to answer/cannot remember	2	18.18%
	Total	11	100.00%

Following the pilot, a few important adjustments were made. Notably, many women experienced difficulty using the tablet, despite the explanations in the “introductory message” recording. As a result, we adjusted our procedure to require a field staff member to guide each participant through the introductory section of the ACASI. Staff were also instructed to conduct a brief demonstration and confirm the participant’s understanding of how to use the device before allowing her to proceed independently with the questionnaire.

## Post-pilot review and ACASI refinement

A review of the initial 198 participants provided valuable insights into the performance of the ACASI tool. While the comprehensive results will be presented alongside the main survey findings, the specific refinements made based on this preliminary data are detailed below.

1. The question “Have you ever had sexual intercourse?” was likely misunderstood as a query about recent sexual activity. Notably, 29% of participants (Fig. 3) who reported a history of pregnancy also indicated they had "never" had sex.
  To resolve this discrepancy and improve data quality, the question was replaced with a more descriptive temporal version:
  When was the last time you had sexual intercourse?
    ◦ Today
    ◦ 1 to 3 days
    ◦ In the last week
    ◦ Within the past 4 weeks
    ◦ Within 1 year
    ◦ More than 1 year ago
    ◦ Never had sexual intercourse
    ◦ Prefer not to answer/cannot remember
2. We reordered several questions after noticing that certain items were influencing how participants answered the ones that followed. For example, we initially placed a question about extramarital partners directly before a question about the total number of partners in the last year. This likely caused "priming bias," where the first question made participants feel judged or uncomfortable, leading them to give less honest answers on the second. By separating these topics, we created a more neutral flow that encourages more accurate reporting.
3. The sequence of response options was reordered to assess whether participants were providing considered responses rather than defaulting to the first or socially desirable option. For example, for the question, *“Who was the person you first had sexual intercourse with?”*, the initial ordering placed “Husband”—a socially preferred response—at the top.
  To minimise this bias and ensure participants listened carefully to all options, the response categories were rearranged, with “Husband” moved lower in the list. This approach aimed to improve the validity of responses by reducing positional and social desirability bias.
  These refinements strengthened both the clarity and accuracy of the survey.

As part of the internal validation process, we deliberately repeated a few similar questions at different points in the ACASI. Spacing these items apart allowed us to assess the consistency of participants’ responses for the interview, thereby strengthening the internal validity of the data and helping to distinguish genuine responses from random or patterned answering.

## Preliminary survey implementation results

**Fig. 4:**
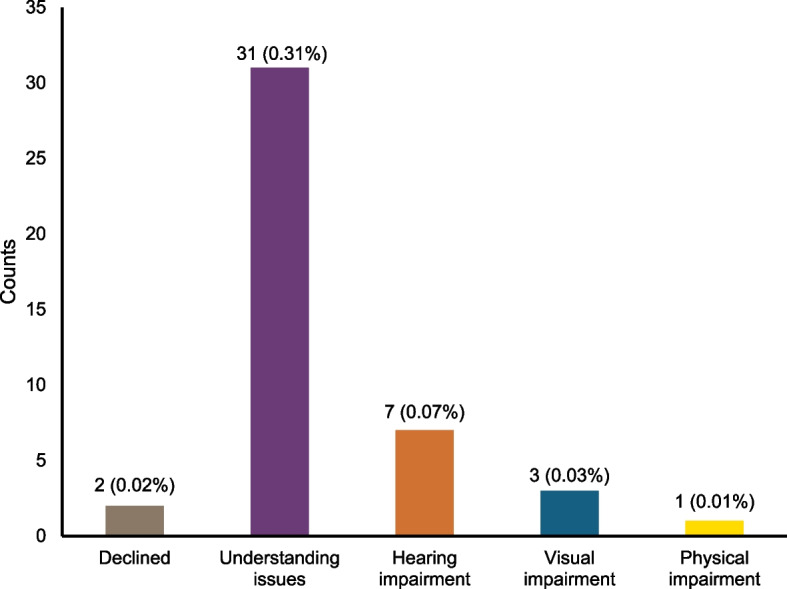
Reasons for ACASI non-completion among the participants who did not complete it (*N* = 10,051)

As of September 2025, close to 10,000 participants have been enrolled in the PHASE survey. Among these, only 44 (< 1%) were unable to complete the ACASI. Of the 44 participants, only 2 (5%) declined participation, while the majority (31; 70%) experienced challenges related to comprehension and use of the device (Fig. 4).

## Discussion

Discussing STIs and sexual health proved particularly sensitive, with some CAB members initially finding the conversations uncomfortable, even when supported by staff translating into local languages. Over time, through repeated dialogue and reassurance, members gradually became more at ease and willing to engage, highlighting both the cultural sensitivity of sexual behaviour research and the importance of building trust and patience in the process. Engagement with CAB members highlighted that community members were generally willing to discuss sexual health issues when assured of confidentiality, particularly if the discussion was not with close acquaintances. In this close-knit setting, where many individuals know each other, the ACASI tool was considered highly appropriate for collecting sensitive information.

But creating an ACASI system for a low-literacy population required careful consideration to ensure usability. The challenge was not only limited to reading ability, but also to participants’ unfamiliarity with information technology, such as navigating touchscreens, using headphones, or following audio prompts. Many participants were encountering digital tools for the first time, which meant that even simple actions like pressing buttons or scrolling through options required step-by-step guidance and practice. The design therefore prioritised intuitive interfaces, clear visual cues, and culturally relevant examples to minimise confusion and ensure participants could engage with the tool confidently, regardless of prior exposure to technology. The “Introduction” section of the ACASI tool, introduced following the pilot experience, where our staff guides and confirms the participant’s understanding of the tool, has proven to be very helpful.

The post-pilot review enabled us a to assess a larger number of participants that revealed some trends. The refinements done following this review, has helped improve clarity and possibly limit social desirability bias.

Implementation results shows a high completion rate, demonstrating the effectiveness of the strategies used to enhance the tool’s usability and acceptability. While we designed the ACASI tool to reduce social desirability bias, some residual cultural barriers may still have influenced the completeness and accuracy of certain responses. For example, the proportion of “Prefer not to answer/cannot remember”, on a glance seems to be higher for some certain sensitive questions. We specifically included this response to track reluctance, and we will analyze these trends in the main survey to measure the impact of sensitive questions on data accuracy.

The survey remains ongoing, with final results expected in mid-2026, at which point the utility of the ACASI tool can be fully assessed. Nonetheless, interim experience indicates encouraging levels of acceptance and response quality.

While this tool significantly enhances data collection, we acknowledge it may not entirely eliminate social desirability bias. However, the insights gained throughout this design process underscore a broader truth: the success of ACASI systems depends on more than just technology. By prioritizing cultural sensitivity, rigorous preparation, and deep community involvement, we have created a framework that respects the participant while safeguarding data integrity – a vital step toward successfully capturing sensitive information in low-literacy, conservative settings.

Click on the link below to view a short snippet of the videos recorded directly from the mobile tablets (https://www.surveycto.com/product/case-studies/how-lshtm-used-surveycto-acasi-in-the-gambia/).

## Data Availability

No datasets were generated or analysed during the current study.

## Abbreviations

ACASI: Audio Computer-Assisted Self-Interviewing
AIDS: Acquired Immunodeficiency Syndrome
AMR: Antimicrobial Resistance
CAB: Community Advisory Board
HIV: Human Immunodeficiency Virus
HPV: Human Papillomavirus
HPV-DNA: Human Papillomavirus DNA
LGA: Local Government Area
LMICs: Low- and Middle-Income Countries
PHASE: Prevalence of Human Papillomavirus, Antimicrobial Resistance, and Sexually Transmitted Infection Estimation
STI: Sexually Transmitted Infection
SurveyCTO: Survey Collect, Transform, and Optimize
URR: Upper River Region
